# Dual single-site catalyst promoter boosts catalytic performance

**DOI:** 10.1093/nsr/nwaa272

**Published:** 2020-10-31

**Authors:** Bin Liu

**Affiliations:** School of Chemical and Biomedical Engineering, Nanyang Technological University, Singapore

Single-atom catalysts (SACs) have received great research interest because of their maximum atom-utilization efficiency and unique catalytic properties [1–5]. However, most of the reported SACs only focus on single-site active components, with rare reports studying catalyst promoters in their single-site forms [6–8]. Because promoters are essential components in many industrial catalysts, the exploration of the single-site promoters should be of great significance in catalysis, both in fundamental and application research. Similar to SACs, these single-site promoters have structural simplicity and homogeneity, and the synergistic effect on the catalytic reaction should be unique yet clarified.

Very recently, Ligen Wang and Limin Wang at the General Research Institute for Nonferrous Metal (GRINM), and Yongjun Ji and Fabing Su at the Institute of Process Engineering, Chinese Academy of Sciences, reported a new catalyst consisting of atomically dispersed Sn and Zn co-promoters on the CuO surface. As demonstrated, this catalyst exhibited a greatly enhanced promoting effect in the industrially important Rochow reaction for dimethyldichlorosilane synthesis.

The authors employed a facile hydrothermal method to synthesize Sn_1_/CuO with abundant surface Cu vacancies. These cation vacancy defects generated by incorporating single-site Sn could be further utilized to anchor single-site Zn. Direct experimental results proved the successful loading of the two single-site promoters on the CuO surface (Fig. 1). X-ray photoelectron spectroscopy (XPS) measurements gave direct evidence that there existed strong interactions between single-site Sn and Zn promoters in this Zn_1_-Sn_1_/CuO catalyst, leading to a significant increase of the electron density on the Cu atoms in CuO. Density functional theory (DFT) calculations show that on the Sn-doped CuO(110) surface, the formation energy of Cu vacancy is 0.78 eV lower than that on the clean CuO(110), indicating that it is easier to form Cu vacancies in the Sn-doped surface. The calculation results also support that Zn prefers to fill in the nearby Cu vacancies caused by Sn doping to form Sn-Zn pairs.

**Figure 1.**
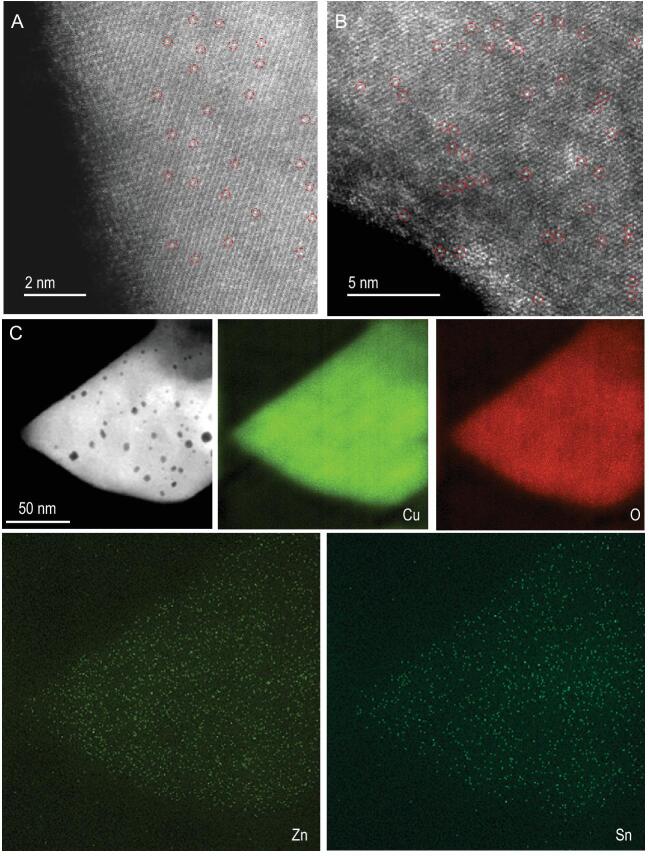
(A) Aberration-corrected high-angle annular dark-field scanning transmission electron microscopy (AC HAADF-STEM) image of Sn_1_/CuO, (B) AC HAADF-STEM and (C) HAADF-STEM images as well as the corresponding energy-dispersive X-ray mappings of Zn_1_-Sn_1_/CuO. The bright dots marked with the red circles in images A and B indicate the single atom. The figure was obtained with permission from Ref. [8].

Compared with conventional catalysts with promoters in the form of nanoparticles, this novel catalyst exhibits much higher activity, selectivity and stability in the synthesis of dimethyldichlorosilane via the industrially important Rochow reaction. The enhanced catalytic performance is attributed to the generated cooperative electronic interaction of single-site Sn and Zn with the CuO support, which further promotes the adsorption of reactant molecules. The authors demonstrated the obvious advantages of the single-site promoters, not only helping to elucidate their real promotion mechanism in the catalytic reaction, but also opening up a new path to optimize catalyst performance. On one hand, the single-site promoters can maximize the catalytic interfaces between the promoter and the catalyst; on the other hand, the specific coordination between the single-site promoters and the catalyst can generate unique electronic properties, thereby promoting the catalytic reaction.

As two or more types of promoters are often used in one industrial catalyst, this work will stimulate research in single-site promoters in catalyst design and thus provide better understanding of the synergistic effect among various promoters. In a certain distance range, the multiple single-site promoters will have strong electronic interactions with the catalyst, which can optimize the electronic structure of the catalyst and thus change its surface adsorption properties. Therefore, it is believed that optimizing the distance between metal atoms is the key to achieving synergy of multiple single-site promoters. Moreover, as the catalyst support is the bridge of electronic interaction, the synergy is expected to be further optimized by adjusting the local microstructure of the catalyst support.

***Conflict of interest statement*.** None declared.

## References

[1] Qiao B Wang AQ Yang XF et al. Nat Chem 2011; 3: 634–41. 10.1038/nchem.1095 21778984

[2] Wei SJ Li A Liu JC et al. Nat Nanotechnol 2018; 13: 856–61. 10.1038/s41565-018-0197-9 30013217

[3] Zhang LL Ren WJ Liu WG et al. Natl Sci Rev 2018; 5: 653–72. 10.1093/nsr/nwy077

[4] Zheng NF Zhang T . Natl Sci Rev 2018; 5: 625. 10.1093/nsr/nwy095

[5] Li ZJ Wang DH Wu YE et al. Natl Sci Rev 2018; 5: 673–89. 10.1093/nsr/nwy056

[6] Wang L Guan EJ Zhang J et al. Nat Commun 2018; 9: 1362. 10.1038/s41467-018-03810-y 29636468 PMC5893533

[7] Cao L Liu W Luo QQ et al. Nature 2019; 565: 631–5. 10.1038/s41586-018-0869-5 30700869

[8] Shi Q Ji YJ Chen WX et al. Natl Sci Rev 2020; 7: 600–8. 10.1093/nsr/nwz196 PMC8288878 34692079

